# Tuberous Sclerosis Complex-Associated Neuropsychiatric Disorders (TAND): New Findings on Age, Sex, and Genotype in Relation to Intellectual Phenotype

**DOI:** 10.3389/fneur.2020.00603

**Published:** 2020-07-07

**Authors:** Petrus J. de Vries, Elena Belousova, Mirjana P. Benedik, Tom Carter, Vincent Cottin, Paolo Curatolo, Maria Dahlin, Lisa D'Amato, Guillaume Beaure d'Augères, José C. Ferreira, Martha Feucht, Carla Fladrowski, Christoph Hertzberg, Sergiusz Jozwiak, John A. Lawson, Alfons Macaya, Ruben Marques, Rima Nabbout, Finbar O'Callaghan, Jiong Qin, Valentin Sander, Matthias Sauter, Seema Shah, Yukitoshi Takahashi, Renaud Touraine, Sotiris Youroukos, Bernard Zonnenberg, John C. Kingswood, Anna C. Jansen, TOSCA Investigators, Nobuo Shinohara

**Affiliations:** ^1^Division of Child and Adolescent Psychiatry, University of Cape Town, Cape Town, South Africa; ^2^Research and Clinical Institute of Pediatrics, Pirogov Russian National Research Medical University, Moscow, Russia; ^3^SPS Pediatrična Klinika, Ljubljana, Slovenia; ^4^TSA Tuberous Sclerosis Association, Nottingham, United Kingdom; ^5^Hôpital Louis Pradel, Claude Bernard University Lyon 1, Lyon, France; ^6^Tor Vergata University Hospital, Rome, Italy; ^7^Astrid Lindgren Childrens Hospital, Stockholm, Sweden; ^8^Novartis Farma S.p.A., Origgio, Italy; ^9^Association Sclérose Tubéreuse de Bourneville, Gradignan, France; ^10^Centro Hospitalar Lisboa Ocidental, Lisbon, Portugal; ^11^Universitätsklinik für Kinder-und Jugendheilkunde, Affiliated Partner of the ERN EpiCARE, Vienna, Austria; ^12^Associazione Sclerosi Tuberosa ONLUS, Milan, Italy; ^13^European Tuberous Sclerosis Complex Association, Dattein, Germany; ^14^Vivantes-Klinikum Neukölln, Berlin, Germany; ^15^Department of Child Neurology, Medical University of Warsaw, Warsaw, Poland; ^16^Department of Neurology and Epileptology, The Children's Memorial Health Institute, Warsaw, Poland; ^17^The Tuberous Sclerosis Multidisciplinary Management Clinic, Sydney Children's Hospital, Randwick, NSW, Australia; ^18^Hospital Universitari Vall d'Hebron, Barcelona, Spain; ^19^Institute of Biomedicine (IBIOMED), University of Leon, León, Spain; ^20^Department of Pediatric Neurology, Necker Enfants Malades Hospital, Paris Descartes University, Paris, France; ^21^Clinical Neurosciences Section, Institute of Child Health, University College London, London, United Kingdom; ^22^Department of Pediatrics, Peking University People's Hospital (PKUPH), Beijing, China; ^23^Tallinn Children Hospital, Tallinn, Estonia; ^24^Klinikverbund Kempten-Oberallgäu gGmbH, Kempten, Germany; ^25^Novartis Healthcare Pvt. Ltd., Hyderabad, India; ^26^National Epilepsy Center, Shizuoka Institute of Epilepsy and Neurological Disorders, NHO, Shizuoka, Japan; ^27^Department of Genetics, CHU-Hôpital Nord, Saint-Étienne, France; ^28^St. Sophia Children's Hospital, Athens, Greece; ^29^University Medical Center, Utrecht, Netherlands; ^30^Cardiology Clinical Academic Group, Molecular and Clinical Sciences Research Centre, St Georges University of London, London, United Kingdom; ^31^Pediatric Neurology Unit, Department of Pediatrics, UZ Brussel VUB, Brussels, Belgium

**Keywords:** intelligence quotient, tuberous sclerosis complex, TSC-associated neuropsychiatric disorders, TOSCA, TAND profile

## Abstract

**Background:** Knowledge is increasing about TSC-Associated Neuropsychiatric Disorders (TAND), but little is known about the potentially confounding effects of intellectual ability (IA) on the rates of TAND across age, sex, and genotype. We evaluated TAND in (a) children vs. adults, (b) males vs. females, and (c) *TSC1* vs. *TSC2* mutations, after stratification for levels of IA, in a large, international cohort.

**Methods:** Individuals of any age with a documented visit for TSC in the 12 months prior to enrolment were included. Frequency and percentages of baseline TAND manifestations were presented by categories of IA (no intellectual disability [ID, intelligence quotient (IQ)>70]; mild ID [IQ 50–70]; moderate-to-profound ID [IQ<50]). Chi-square tests were used to test associations between ID and TAND manifestations. The association between TAND and age (children vs. adults), sex (male vs. female), and genotype (*TSC1* vs. *TSC2*) stratified by IA levels were examined using the Cochran–Mantel–Haenszel tests.

**Results:** Eight hundred and ninety four of the 2,211 participants had formal IQ assessments. There was a significant association (*P* < 0.05) between levels of IA and the majority of TAND manifestations, except impulsivity (*P* = 0.12), overactivity (*P* = 0.26), mood swings (*P* = 0.08), hallucinations (*P* = 0.20), psychosis (*P* = 0.06), depressive disorder (*P* = 0.23), and anxiety disorder (*P* = 0.65). Once controlled for IA, children had higher rates of overactivity, but most behavioral difficulties were higher in adults. At the psychiatric level, attention deficit hyperactivity disorder (ADHD) was seen at higher rates in children while anxiety and depressive disorders were observed at higher rates in adults. Compared to females, males showed significantly higher rates of impulsivity and overactivity, as well as autism spectrum disorder (ASD) and ADHD. No significant age or sex differences were observed for academic difficulties or neuropsychological deficits. After controlling for IA no genotype-TAND associations were observed, except for higher rates of self-injury in individuals with *TSC2* mutations.

**Conclusions:** Findings suggest IA as risk marker for most TAND manifestations. We provide the first evidence of male preponderance of ASD and ADHD in individuals with TSC. The study also confirms the association between *TSC2* and IA but, once controlling for IA, disproves the previously reported *TSC2* association with ASD and with most other TAND manifestations.

## Introduction

Tuberous sclerosis complex (TSC) is a genetic disorder with prevalence of 1:5,800 live births. It is caused by mutation in either the *TSC1* or *TSC2* gene and characterized by the growth of benign hamartomas in multiple organs including the brain, and is often associated with a high rate of neurological deficits (1). Apart from the range of physical manifestations observed, around 90% of patients with TSC exhibit some neuropsychiatric manifestations and these are associated with the greatest burden of care for families (1–5). Although most people with TSC will have neuropsychiatric disorder, only a small proportion typically ever receive screening, diagnosis, and treatment for these (6). The term TAND (TSC-associated neuropsychiatric disorders) was therefore coined to capture the multi-level manifestations, and a TAND Checklist was developed as a simple screening tool to help in the identification and prioritization of TAND manifestations (7, 8).

TAND manifestations are classified into 6 levels including behavioral, psychiatric, intellectual, academic, neuropsychological, and psychosocial levels (3). Among behavioral difficulties, the reported ranges to date include depressed mood (19–43%), anxiety (41–56%), self-injury (17–69%), aggression (37–66%), temper tantrums (47–70%), overactivity/hyperactivity (22–73%), impulsivity (36–62%), and sleep difficulties (15–74%) (6, 9–11). At the psychiatric level, reported rates include autism spectrum disorder (ASD; 40–50%), attention deficit hyperactivity disorder (ADHD; 30–40%), anxiety and depressive disorder (27–56%) and psychosis (2.3%) (1, 6, 9). At the intellectual level, around 40–50% of individuals with TSC are considered to have normal intellectual ability (IA), and the remaining have some degree of intellectual disability (ID) (2, 12, 13). The majority of individuals with TSC have had difficulties in academic or scholastic skills (2). Individuals with TSC are at high risk of a range of neuropsychological deficits including attention deficits, memory deficits, and executive deficits. At the psychosocial level, family stress and difficulties with self-esteem and self-efficacy are often reported (3, 14).

The etiology of TAND manifestations has received some scientific investigation over the last few decades. It is well-established that epilepsy (infantile spasms and other seizure types) is a clear risk marker for many TAND manifestations, particularly intellectual ability (1, 15, 16). The role of structural brain abnormalities such as cortical tubers or SEGA has been less clear (1, 3, 17). Direct molecular models suggesting that the functional consequences of *TSC1* or *TSC2* mutations may directly lead to TAND, and combinatorial models of the above, have also been suggested (1, 18).

Given the relative rarity of TSC, the evidence-base for TAND manifestations and their patterns have, until recently, been based on relatively small-scale studies that typically examined only some of the levels of TAND, and that were typically from a single country. Very little was known about the differences between children and adults or between those with *TSC1* vs. *TSC2* mutations. In a recent study, we evaluated TAND in a large multicenter international study (TOSCA) and examined profiles of manifestations in children vs. adults, in different age-bands, and in those with *TSC1, TSC2*, and no mutation identified (NMI) (2). Findings in the study were based on data from 2,216 participants at the third interim analysis (cut-off 30 September 2015) of the TOSCA natural history study. The study showed significantly higher rates of overactivity and impulsivity in children and higher rates of anxiety, depressed mood, mood swings, obsessions, psychosis, and hallucinations in adults. Individuals with *TSC2* mutations had higher frequency of self-injury, ASD, academic difficulties and neuropsychological deficits, while those with NMI showed a mixed pattern of TAND manifestations. Interestingly, individuals with *TSC1* mutations showed higher rates of impulsivity, anxiety, depressed mood, hallucinations, psychosis, and of ADHD, anxiety and depressive disorders (2).

A key finding from the study was the observation that those with *TSC2* mutations had significantly higher rates of ID. Intellectual ability is known to be a strong correlate or risk marker of behavioral, psychiatric, academic, and neuropsychological deficits both in general population and in individuals with TSC (6, 19). For example, an earlier study in 265 children and adolescents with TSC showed differential rates of many behavioral manifestations, ASD and ADHD, in individuals with and without ID (6). The fundamental role of IA as risk marker for TAND therefore raises concerns about the previous findings of de Vries and colleagues (2) in terms of child vs. adult differences, and about *TSC1* vs. *TSC2* differences in TAND.

It is also well-established that many psychopathologies have been associated with differential rates between male and females. For example, boys and men are typically associated with higher rates of ASD and ADHD, while girls and women are typically associated with higher rates of anxiety and mood disorders (20–24). Studies in TSC to date have shown conflicting findings in relation to sex differences of TAND. In one small study from Wessex, UK a significant male preponderance in the rates of ID was reported (25). In contrast, other studies have shown no difference in the rates of behavioral problems, psychiatric disorders or ID (6, 26). To date no studies have compared academic/scholastic difficulties and neuropsychological deficits between male and female individuals with TSC.

Here, we therefore set out to perform a detailed exploration of the association of TAND manifestations (a) between children and adults, (b) between males and females, and (c) between those with *TSC1* and *TSC2* mutations, in a large international sample of individuals with TSC, stratified for their levels of IA. We hypothesized that, after controlling for levels of IA (a) the significant differences observed between children and adults would be maintained (2), (b) that, as per previous TSC research no sex differences would be observed in TAND (6, 26), and (c) that the *TSC1-TSC2* differences observed in our earlier study would be maintained (2).

## Participants and Methods

TOSCA, a multicenter, international study in individuals with TSC, was conducted at 170 sites in 31 countries. The study methodology of TOSCA has been detailed previously (27). In brief, the study consisted of a core section and 6 ancillary research projects, focusing each on subependymal giant cell astrocytomas (SEGA), renal angiomyolipoma and lymphangiomyomatosis, genetics, TAND, epilepsy, and quality of life. TAND data were collected from retrospective and prospective information available to study clinicians using a standardized data recording sheet as part of the case report form (CRF). The TAND data recording sheet were a precursor of the TAND Checklist (8). Comprehensive data were collected at baseline and annually thereafter for up to 5 years. Interim analyses of all data collected were done annually. Here we present results of the final analysis (last patient last visit, 10 August 2017).

All TOSCA participants in the final analysis with formal IQ assessment data were included in this study. Frequency and percentages of baseline TAND manifestations were presented by categories of IA [intelligence quotient (IQ) >70 = no ID (noID); IQ = 50–70 = mild ID (MID); IQ <50 = moderate-to-profound ID (M-PID)]. Chi-square test was used to examine the association between ID and TAND manifestations. The association between TAND and age [children [aged ≤18 years] vs. adults [aged >18 years]], sex (male vs. female), and genotype (*TSC1* vs. *TSC2*) stratified by IA (noID, MID, M-PID) was examined using the Cochran–Mantel–Haenszel tests. Statistical significance was set at *p* < 0.05.

The study was designed and conducted in accordance with the Good Clinical Practice principles, the Declaration of Helsinki, and all the local regulations. The Institutional Review Board or Ethics Committee at each participating center approved all the TOSCA related documents. Written informed consent was obtained from all participants, parents, or guardians prior to enrolment.

## Results

Overall 2,214 participants with TSC were enrolled into the TOSCA registry from 170 sites across 31 countries. Of these, data of 2,211 eligible participants were analyzed. Data of 3 participants were excluded from the analysis due to major protocol deviations. Of the 2,211 participants, 894 (40.4%) had formal IQ assessments; 395 had normal IQ, 251 had MID and 248 had M-PID. Baseline demographics of this cohort were similar to that of the overall cohort and those without IQ (Table 1).

**Table 1:** Demographics of participants in the TOSCA study.

**Characteristics**	**Overall Cohort (*N* = 2,211)**	**Participants with IQ assessments (*N* = 894)**	**Participants without IQ assessments (*N* = 1,305)**
Age at TSC diagnosis,^a^ years, median (range)	1.0 (0–69)	1.0 (0–60)	1 (0–69)
Gender, *n* (%)			
Males	1059 (47.9)	432 (48.3)	621 (47.6)
Females	1152 (52.1)	462 (51.7)	684 (52.4)
Genetic molecular testing performed, *n* (%)	1011 (45.7)	468 (52.3)	543 (41.6)
Genetic testing, *n* (%)			
No mutation identified	148 (14.6)	69 (14.7)	79 (14.5)
*TSC1* mutation	191 (18.9)	94 (20.1)	97 (17.9)
*TSC2* mutation	649 (64.2)	301 (64.3)	348 (64.1)
Both *TSC1* and *TSC2* mutation	5 (0.5)	0	5 (0.9)
Data not available	18 (1.8)	4 (0.8)	14 (2.6)
Mutation variation type^b^, *n* (%)			
Only pathogenic mutation	663 (65.6)	331 (70.7)	332 (61.1)
Only variant of unknown significance	43 (4.3)	18 (3.8)	25 (4.6)
Time from TSC diagnosis to molecular testing, months, mean (SD)	81.8 (116.58)	84 (99.84)	79.8 (129.78)
Participants with prenatal diagnosis, *n* (%)	154 (7.0)	64 (7.2)	90 (6.9)
Participants with biological parent diagnosed with TSC, *n* (%)			
Mother	184 (19.5)	95 (18.3)	98 (21.4)
Father	130 (15.7)	63 (14.9)	67 (16.6)

*IQ, intelligence quotient; SD, standard deviation; TSC, tuberous sclerosis complex*.

a *Data available for 2,054 participants in the overall cohort*.

b *The count (n) also includes 23 participants who had both mutation types*.

### Overall TAND Manifestations and Their Association With Levels of Intellectual Ability (IA)

The overall and stratified frequencies of TAND manifestations in the final TOSCA cohort are depicted in Table 2. The majority of behavioral difficulties showed significant association (*P* < 0.05) with the levels of IA, except impulsivity (*P* = 0.12), overactivity (*P* = 0.26), mood swings (*P* = 0.08), hallucinations (*P* = 0.20), and psychosis (*P* = 0.06, Table 2). IA showed a significant association with ASD, ADHD, and other psychiatric disorders, but not with depressive disorder (*P* = 0.23) or anxiety disorder (*P* = 0.65). Academic difficulties and neuropsychological deficits were significantly associated with levels of IA (Table 2).

**Table 2:** TAND manifestations in all participants with available IQ data stratified by levels of intellectual ability (noID [IQ>70], MID [IQ 50–70] and M-PID [IQ<50]).

**TAND manifestation**	**All participants with**	**Level of intellectual ability**			***P*-value^a^**
	**IQ data available**	**NoID (*n* = 395)**	**MID (*n* = 251)**	**M-PID (*n* = 248)**	
	**(*N* = 894)**	***n* (%)**	***n* (%)**	***n* (%)**	
	***n* (%)**				
**Behavioral level**					
Sleep difficulties	172 (40.3)	46 (31.9)	45 (34.9)	81 (52.6)	0.0004
Severe aggression	100 (23.3)	22 (15.6)	37 (27.2)	41 (26.8)	0.03
Self-injury	63 (14.7)	8 (5.7)	14 (10.6)	41 (26.1)	<0.0001
Impulsivity	201 (47.2)	57 (40.7)	70 (53.0)	74 (48.1)	0.12
Overactivity	191 (44.4)	55 (39.0)	65 (48.5)	71 (45.8)	0.26
Depressed mood	76 (18.3)	37 (26.1)	27 (21.3)	12 (8.2)	0.0003
Anxiety	146 (34.9)	56 (40.0)	54 (40.3)	36 (25.0)	0.009
Mood swings	134 (32.3)	36 (26.3)	50 (39.1)	48 (32.0)	0.08
Obsessions	71 (17.1)	10 (7.2)	26 (20.0)	35 (24.1)	0.0004
Hallucinations	18 (4.3)	5 (3.5)	9 (7.0)	4 (2.8)	0.20
Psychosis	25 (6.0)	3 (2.1)	11 (8.3)	11 (7.6)	0.06
**Psychiatric level**					
Autism spectrum disorder (ASD)	165 (21.0)	14 (4.0)	31 (14.2)	120 (55.6)	<0.0001
Attention deficit hyperactivity disorder (ADHD)	167 (22.2)	56 (16.0)	55 (25.5)	56 (29.9)	0.0004
Depressive disorder	42 (5.7)	23 (6.7)	13 (6.3)	6 (3.2)	0.23
Anxiety disorder	87 (11.7)	38 (11.0)	28 (13.5)	21 (11.1)	0.65
Other psychiatric disorder	61 (8.2)	17 (4.9)	20 (9.6)	24 (12.6)	0.005
**Academic level**					
Participants with academic/scholastic difficulties	450 (68.0)	143 (47.2)	156 (82.5)	151 (88.8)	<0.0001
Participants assessed for difficulties	290 (76.9)	96 (75.0)	103 (79.8)	91 (75.8)	0.62
**Neuropsychological level**					
Participants assessed for neuropsychological skills	408 (58.1)	183 (56.5)	123 (60.9)	102 (58.0)	0.61
Participants with any deficit (Performance <5th percentile)	250 (69.6)	69 (41.3)	92 (90.2)	89 (98.9)	<0.0001

*Values are expresses as number (%). Percentages are calculated excluding missing/unknown data*.

*IQ, intelligence quotient; noID, no intellectual disability; MID, mild intellectual disability; M-PID, moderate-to-profound intellectual disability; TAND, tuberous sclerosis complex-associated neuropsychiatric disorders*.

a *P-value calculated from chi-square to test the association between categories of intellectual disability (NoID, MID and M-PID) and presence of respective TAND manifestation*.

### TAND Manifestations in Children vs. Adults Stratified by Intellectual Ability (IA)

**Figure 1:**
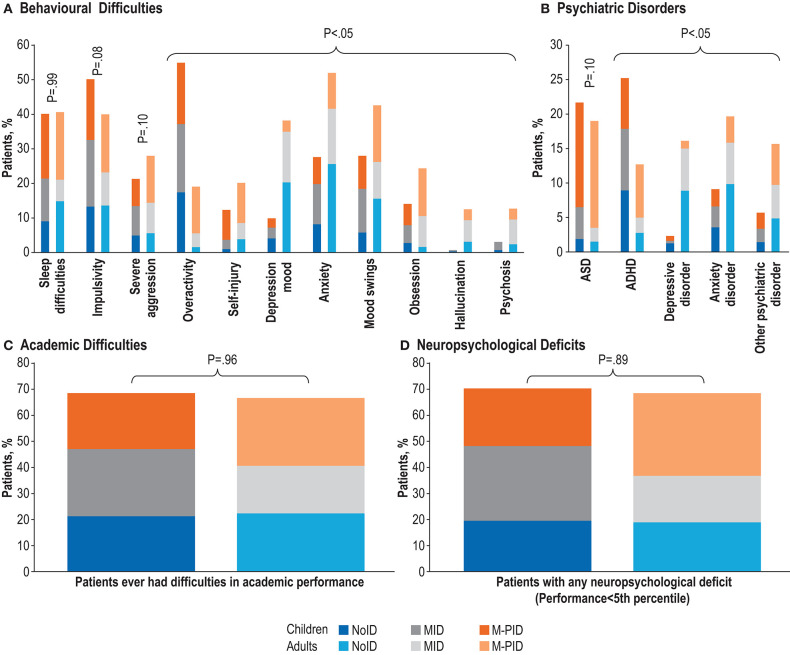
Frequency of TAND features stratified by levels of intellectual ability (noID [IQ>70], MID [IQ 50–70] and M-PID [IQ<50]) in children vs. adults. **(A)** Behavioral difficulties. **(B)** Psychiatric disorders. **(C)** Academic difficulties. **(D)** Neuropsychological deficits. Percentages calculated excluding missing/unknown data.

Once controlled for IA, adults showed significantly higher rates of most behavioral difficulties in comparison to children (*P* < 0.05), including severe aggression, self-injury, anxiety, mood swings, hallucination, obsession, and psychosis. Children showed significantly higher rates only of overactivity (*P* < 0.05, Figure 1A). No differences were observed between children and adults on sleep difficulties (*P* = 0.99), impulsivity (*P* = 0.08) or severe aggression (*P* = 0.10). At the psychiatric level, the rate of ASD (*P* = 0.10) was not significantly different between children and adults (Figure 1B). In contrast, ADHD (*P* < 0.05) were seen at higher rates in children, while anxiety disorders, depressive disorders and other psychiatric disorders were observed at higher rates in adults. No significant differences were seen in the rates of academic difficulties (Figure 1C) or neuropsychological deficits (Figure 1D) between children and adults in IQ-stratified groups (Supplementary Table 1).

### TAND Manifestations in Males vs. Females Stratified by Intellectual Ability (IA)

**Figure 2:**
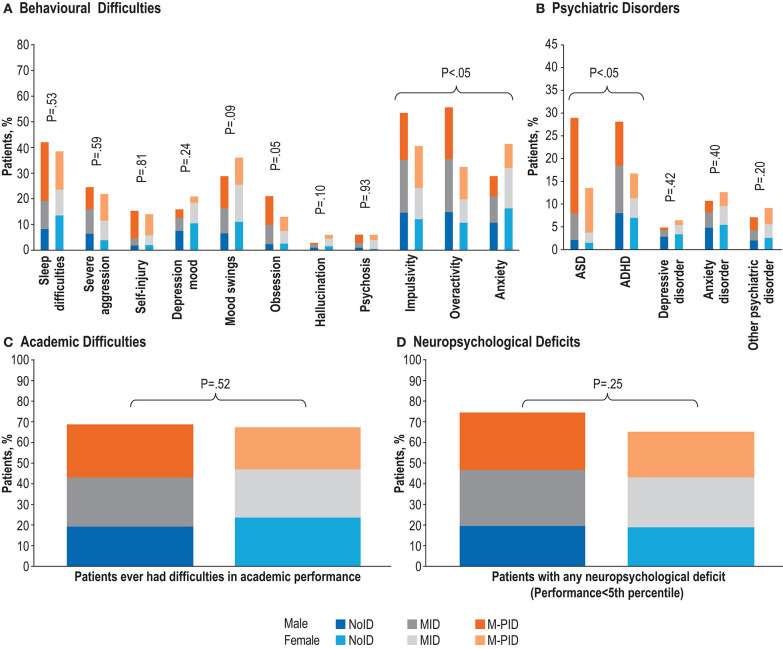
Frequency of TAND features stratified by levels of intellectual ability (noID [IQ>70], MID [IQ 50–70] and M-PID [IQ<50]) in male vs. female. **(A)** Behavioral difficulties. **(B)** Psychiatric disorders. **(C)** Academic difficulties. **(D)** Neuropsychological deficits. Percentages calculated excluding missing/unknown data.

Two behavioral manifestations (impulsivity and overactivity) were seen at significantly higher rates in males than females, while anxiety rates were higher in females (Figure 2A, Supplementary Table 2). No other behavioral manifestations were statistically significantly different between males and females once controlled for IA. At the psychiatric level, ASD and ADHD were seen at significantly higher rates in males than females, but depressive, anxiety and other psychiatric disorders were not significantly different (Figure 2B). No differences were observed between males and females in academic difficulties (Figure 2C) or neuropsychological deficits (Figure 2D).

### TAND Manifestations in *TSC1* vs. *TSC2* Stratified by Intellectual Ability (IA)

**Figure 3:**
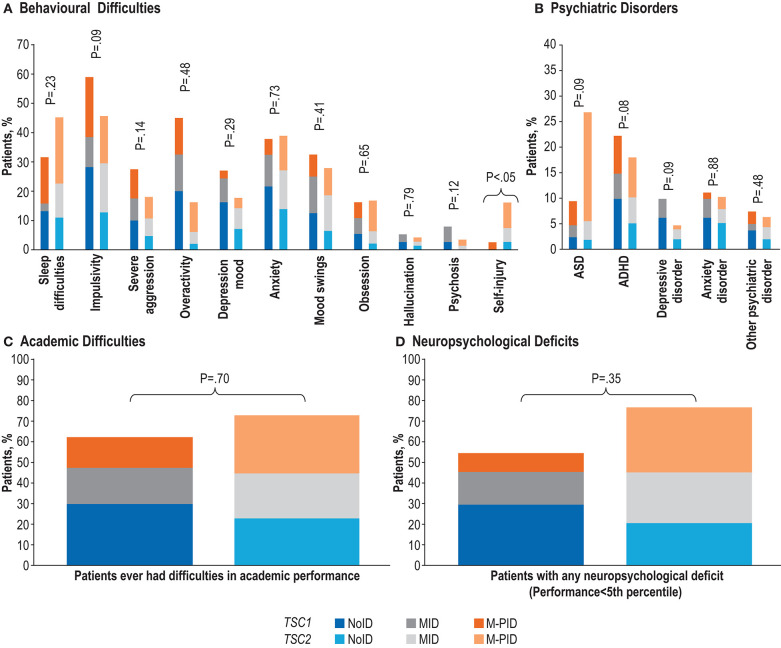
Frequency of TAND features stratified by levels of intellectual ability (noID [IQ>70], MID [IQ 50–70] and M-PID [IQ<50]) in *TSC1* vs. *TSC2*. **(A)** Behavioral difficulties. **(B)** Psychiatric disorders. **(C)** Academic difficulties. **(D)** Neuropsychological deficits. Percentages calculated excluding missing/unknown data.

After controlling for levels of IA, only one of all the TAND manifestations (self-injury) was observed at significantly higher rates in patients with *TSC2* mutations vs. those with *TSC1* mutations. No genotype-TAND associations were seen on any other behavioral manifestations (Figure 3A, Supplementary Table 3), psychiatric disorders (Figure 3B), academic difficulties (Figure 3C) or neuropsychological deficits (Figure 3D). In particular, the previously reported association between *TSC2* mutations and ASD was not statistically significant (*P* = 0.09).

## Discussion

In this study we set out to examine TAND manifestations in relation to age, sex, and genotype in an IA-stratified sample of individuals from 31 countries. The large-scale cohort allowed us to perform analyses not previously possible. In the overall cohort of 894 participants who had formal IQ evaluations, IA was significantly associated with the majority of behavioral manifestations, apart from impulsivity, overactivity, mood swings, hallucinations, and psychosis. In a similar pattern at the psychiatric level, IA was associated with ASD, ADHD, and other psychiatric disorders, but not with depressive disorders or anxiety disorders. Academic difficulties and neuropsychological deficits showed a clear association with the levels of IA.

In terms of differences between children and adults, we predicted that all age-related TAND manifestations previously observed (2) would be maintained in stratified groups. In the earlier study overactivity, impulsivity and ADHD were more prominent in children, while anxiety, mood swings, depressed mood, psychosis, hallucinations, depressive disorder, and anxiety disorder were more prominent in adults. After controlling for IA, only overactivity was observed at significantly a higher rate in children, while most other behavioral manifestations had higher rates in adults. These observations challenge previous data that suggested an improvement or reduction in behavioral difficulties in individuals with TSC over time. In keeping with general population patterns, even after IA stratification, ADHD was observed at higher rates in children, and depressive and anxiety disorders at higher rates in adults. No academic difficulties or neuropsychological deficits showed age-based patterns after stratification. Mindful of the fact that these findings are based on cross-sectional rather than longitudinal data, our results suggest the need for careful longitudinal examination of behavioral change and emergence of psychopathology over time in TSC.

We predicted that, based on previous TSC research (6, 26), no sex differences would be observed. Contrary to the hypothesis, impulsivity, overactivity, anxiety, and obsessions, as well as ASD and ADHD were significantly more common in males. These observations are therefore the first clear evidence of a sex-related preponderance of ASD, ADHD and related behavioral manifestations in TSC. Anxiety symptoms were observed at higher rates in females, but, interestingly, no sex differences were observed in rates of anxiety disorders. Findings suggest that, at least for some psychopathologies in TSC, sex may play a contributory role. Future research should therefore consider the potential role of sex alongside genetic and other environmental factors in the pathway to psychopathology in TSC. Our results certainly highlight the need to control for sex in any comparative studies involving individuals with TSC.

Given previous reports of an association between *TSC2* and more severe TSC manifestations, we predicted the same pattern for TAND. We observed a clear correlation between levels of IA and genotype, with *TSC2* more likely to be associated with ID. However, after controlling for levels of IA, only one of all the genotype-TAND correlations was statistically significant (self-injury, *P* = 0.0496). We are cautious not to over-interpret what might have been a spurious finding. Importantly, the previously suggested association between *TSC2* mutations and ASD was not replicated in our data. These results support the previous evidence of the strong association between levels of intellectual ability and psychopathologies in the general population (28, 29), and provide the first clear evidence of the association between IA and all levels of TAND investigated here. However, our findings did not suggest a specific association between *TSC1* or *TSC2* and TAND once levels of IA had been controlled for. Our findings therefore underline the importance of controlling for the levels of IA in any future study that may wish to compare or contrast TAND in individuals with *TSC1* and *TSC2* mutations.

Overall our findings underline the prominent role of IA as a risk marker for TAND manifestations, illustrated the differences in TAND profiles between children and adults over and above IA, and, for the first time, identified male sex as an additional risk marker for TAND. Together, these highlight the need always to consider intellectual ability, age, and sex in any TAND-related research investigation.

### Implications for Clinical Practice

The findings reported here support the value of an intellectual ability evaluation of all individuals with TSC. Even though we reported the largest cohort with formal IQ assessments to date (*n* = 894), this represented only 40.4% of the overall TOSCA cohort. Even in expert TSC centers, IQ was therefore not routinely evaluated. With regards to age-related changes, overactivity showed lower rates in adults, but the majority showed higher rates in adults stratified by IA. It will be important not to interpret this as “worsening” of behaviors in adults with TSC given that our dataset was cross-sectional. Longitudinal studies will be important to examine this aspect, but, for clinical practice, results suggest that not all behavioral manifestations may always improve. The clear increase in mood and anxiety symptoms and disorders into adulthood emphasizes the dynamic nature of TAND, and underlines the importance of annual screening for TAND using tools such as the TAND Checklist, as recommended in the International Consensus Guidelines (8, 30). The sex differences observed with higher rates of ASD and ADHD in males with TSC are in keeping with general population observations, and raise interesting scientific questions. From a clinical perspective, even though some sex differences were observed, it is also clear that all males and females should be monitored for all TAND manifestations. At a clinical level the absence of genotype-TAND correlations suggests that, apart from the greater likelihood of ID in association with *TSC2*, clinicians should not suggest to families to expect significantly different TAND profiles in an individual with *TSC1* vs. *TSC2*. All individuals with TSC should therefore be screened and monitored for all TAND manifestations throughout their lifespan.

### Limitations

We acknowledge the limitations intrinsic to a large-scale, international, non-interventional/observational study. These included the fact that participants were recruited from expert TSC centers around the world, included evaluation in a range of languages, and the fact that evaluations were performed based on standard clinical practice in each center, rather than on a pre-specified set of evaluation instruments. However, these limitations are, at least in part, off-set by the large-scale and “real-world” nature of the cohort across multiple centers and countries. We acknowledge the high proportion of non-reported (missing) data by sites, including IA evaluation on only 40.4% of the cohort. This finding emphasizes that, even in expert TSC centers, TAND manifestations are often not examined and therefore not treated. We also acknowledge that we focused here on the association between intellectual ability, age, sex, and genotype and that we did not include the potential contributions of physical risk markers (e.g., seizures, SEGA or other TSC manifestations) into our modeling of associations.

## Conclusion

The TOSCA study confirmed the association between levels of IA and TAND manifestations, suggesting IA as risk marker for most TAND manifestations and provided the first evidence of a male preponderance of ASD and ADHD in individuals with TSC. The study also confirmed the association between *TSC2* and IA but disproved the previously reported *TSC2* association with ASD and most other TAND manifestations once controlled for IA. Overall, the study reinforces the high frequency of TAND manifestations in all individuals with TSC across age, sex, and genotype, and strengthens the evidence-base for regular screening, comprehensive evaluation and intervention for the dynamic and variable range of neuropsychiatric manifestations associated with TSC.

## Data Availability Statement

Novartis supports the publication of scientifically rigorous analysis that is relevant to patient care, regardless of a positive or negative outcome. Qualified external researchers can request access to anonymized patient-level data, respecting patient informed consent, contacting study sponsor authors. The protocol can be accessed through EnCePP portal http://www.encepp.eu/ (EU PAS Register Number EUPAS3247).

## Ethics Statement

The study was designed and conducted in accordance with the Good Clinical Practice principles, the Declaration of Helsinki, and all the local regulations. The Institutional Review Board or Ethics Committee at each participating center approved all the TOSCA related documents. Written informed consent was obtained from all participants, parents, or guardians prior to enrolment.

### List of Ethics Committees

The study protocol and all amendments were reviewed and approved (if applicable) by independent Ethics Committee/Institutional Review Board for each centre: National Hospital Organization Central Ethics Committee; Gazi University Clinical Research Ethics Committee; Independent Multidisciplinary Committee on Ethical Review of Clinical Trials; Peking Union Medical College Hospital; Commissie Medische Ethiek UZ Brussel; CNIL (Commission National de l'Informatique et des Libertés), CCTIRS (Comité Consultatif sur le traitement de l'information en matière de recherche dans le domaine de la santé); Comité Etico Investigación Clínica de Euskadi (CEIC-E); Consejeria de Salud y Bienestar Social, Dirección General de Calidad, Investigación, Desarrollo e Innovación, Comité Coordinador de Ética de la Investigación Biomédica de Andalucía; Research Ethics Committee of the University of Tartu (UT REC); Ethikkommission der Medizinischen Universität Graz; North Wales REC – West; Regionala Etikprövningsnämnden i Göteborg; REK – Regionale komiteer for medisinsk og helsefaglig forskningsetikk; Komisja Bioetyczna przy Instytucie “Pomnik Centrum Zdrowia Dziecka”; Ethikkommission bei der Ludwig-Maximilians-Universitat München; Hokkaido University Hospital Independent clinical research Institutional Ethics Committee; Medical Juntendo University Institutional Ethics Committee; National Center for Chile Health and Deveropment of IRB; Osaka University Hospital of IRB; Ethics Committee at Moscow Institute of Pediatrics and Pediatric Surgery; Peking University First Hospital; Sanbo Brain Hospital Capital Medical University; Tianjin Children's Hospital; Childrens Hospital of Fudan University; Zhongshan Hospital Fudan University; Fudan University Shanghai Cancer Center; The Second Affiliated Hospital of Guangzhou Medical University; The First Affiliated Hospital, Sun Yan-sen University; The First Affiliated Hospital of Guangzhou Medical University; Shenzhen Children's Hospital; West China Hospital, Sichuan University; Xijing Hospital; Children's Hospital of Chongqing Medical University; Wuhan Children's Hospital; The Second Affiliated Hospital of Xi'an Jiaotong University; Guangdong 999 Brain Hospital; Seoul National University Hospital Institutional Review Board; National Taiwan University Hospital (NTUH) Research Ethics Committee (REC); Institutional Review Board of the Taichung Veterans General Hospital; Institutional Review Board of Chung Shan Medical University Hospital; Institutional Review Board, Tungs' Taichung MetroHarbor Hospital; Institutional Review Board of National Cheng Kung University Hospital; Metro South Human Research Ethics Committee; Sydney Children's Hospital Network Human Research Ethics Committee; St Vincents Hospital Human Research Ethics Committee; Royal Melbourne Hospital Human Research Ethics Committee; Siriraj Institutional Review Board; The Institutional Review Board, Faculty of Medicine, Chulalongkorn University, King Chulalongkorn Memorial Hospital; The Committee on Human Rights Related to Research Involving Human Subjects; Institutional Review board, Royal Thai Army Medical Department IRB RTA, Phramongkutklao College of Medicine; Research Ethics Committee, Faculty of Medicine, Chiang Mai University; Research and Development, Queen Sirikit National Institute of Child Health; Human Research Ethics Committee, Faculty of Health Sciences, University of Cape Town; Shaare Zedek Meidcla Center Helsinki Committee; Sheba Medical Center Helsinki Committee; Tel Aviv Sourasly Medical Center Helsinki Committee; General University Hospital of Patras Ethics Committee; Pendeli Children's Hospital Ethics Committee; General University Hospital of Athens “G. Gennimatas” Ethics Committee; Evaggelismos General Hospital Ethics Committee; General University Hospital of Thessaloniki “AHEPA” Ethics Committee; General University Hospital of Ionnina Ethics Committee; METC UMC Utrecht; Direcció General de Regulació, Planificació i Recursos Sanitaris; Comité Ético de Investigación Clínica del Hospital Universitario Vall d'Hebron de Barcelona, Generalitat de Catalunya, Departament de Salut; Comité Ético de Investigación Clínica Hospital Universitario La Paz; Dirección General de Ordenación e Inspección, Consejería de Sanidad Comunidad de Madrid, Servicios de Control Farmacéutico y Productos Sanitarios; Comité Etico Investigación Clínica del Hospital Universitario y Politécnico de La Fe; Dirección General de Farmàcia i Productes Sanitaris, Generalitat de Valencia; Comité de Ética de la Investigación de Centro de Granada; Instituto Aragonés de Ciencias de la Salud (IACS); Comité Etico Investigación Clínica Regional del Principado de Asturias; Comité Etico Investigación Clínica Hospital 12 de Octubre; Comité Etico Investigación Clínica Hospital Universitario Virgen de la Arrixaca; Sección de Ordenación e Inspección Farmacéutica Departamento de Salud; Comité Ético de Investigación Clínica del Hospital Universitario del Río Hortega de Valladolid; Comissão de Ética para a Saúde (CES), Centro Hospitalar de Lisboa Ocidental, EPE; Comissão de Ética para a Saúde (CES), Centro Hospitalar do Porto, EPE; Comissão de Ética para a Saúde (CES), Centro Hospitalar Lisboa Central, EPE; Comissão de Ética para a Saúde (CES), Hospital Garcia de Orta, EPE; Comissão de Ética para a Saúde (CES), Centro Hospitalar de São João, EPE; Comissão de Ética para a Saúde (CES), Hospital Professor Doutor Fernando Fonseca, EPE; Comissão de Ética para a Saúde (CES), Centro Hospitalar do Algarve, EPE (Unidade de Faro); LUHS Kaunas Regional Biomedical Research Ethics Committee; Paula Stradiņa klīniskās universitātes slimnīcas, Attīstības biedrības Klīniskās izpētes Ētikas komiteja, Ethics Committee for Clinical Research; Komisija Republike Slovenije za medicinsko etiko; Comitato Etico Indipendente Presso La Fondazione Ptv Policlinico Tor Vergata Di Roma; Comitato Etico Regione Calabria Sezione Centro c/o A.O.U. Mater Domini Di Catanzaro; Comitato Etico Azienda Ospedaliera Universitaria Di Cagliari; Comitato Etico Cardarelli-Santobono c/o Ao Cardarelli; Comitato Etico Per La Sperimentazione Clinica Delle Province Di Verona E Rovigo, Presso Aoui Verona; Eticka Komise Fn Brno; Eticka Komisia Dfnsp Bratislava; Eticka Komisia Pri Dfn Kosice; Eticka Komisia Bratislavskeho Samospravneho Kraja; Comisia Naţională de Bioetică a Medicamentului şi a Dispozitivelor Medicale; Comitato Etico Milano area 1 c/o ASST FBF Sacco – P. O. L. Sacco; Comité de Ética de la Investigación de Centro Hospital Universitario Virgen del Rocío; Comité Ético de Investigación Clínica Fundació Sant Joan de Déu Generalitat de Catalunya, Departament de Salut; Comité Ético de Investigación Clínica Hospital Infantil Universitario Niño Jesús; Consejería de Sanidad Dirección General de Salus Pública Junta de Castilla León; Dirección General de Asistencia Sanitaria, Consejería de Sanidad Gobierno del Principado de Asturias; Dirección General de Planificación, Ordenación Sanitaria y Farmacéutica e Investigación, Consejeria de Sanidad y Política Social Región de Murcia; Ethics Committee at Moscow Institute of Pediatrics and Pediatric Surgery; Paula Stradiņa klīniskās universitātes slimnīcas, Attīstības biedrības Klīniskās izpētes Ētikas komiteja, Ethics Committee for Clinical Research; The First Affiliated Hospital of The Fourth Military Medical University; Zhongshan Hospital Fudan University.

## Author Contributions

PV, TC, VC, GB, CF, FO'C, JQ, YT, and SY designing the study, data interpretation, drafting, revising, final review, and approval of the manuscript. EB, MB, PC, MD, JF, MF, CH, SJ, JK, JL, AM, RN, VS, MS, RT, BZ, and AJ designing the study, patient accrual, clinical care, data interpretation, drafting, revising, final review, and approval of the manuscript. LD'A designing the study, trial management, data collection, data analysis, data interpretation, drafting, revising, final review, and approval of the manuscript. RM designing the study, data analysis, data interpretation, drafting, revising, final review, and approval of the manuscript. SS designing the study, trial statistician, data analysis, data interpretation, drafting, revising, final review, and approval of the manuscript. All authors contributed to the article and approved the submitted version.

### TOSCA Investigators

Japan: Nobuo Shinohara, Shigeo Horie, Masaya Kubota, Jun Tohyama, Katsumi Imai, Mari Kaneda, Hideo Kaneko, Yasushi Uchida, Tomoko Kirino, Shoichi Endo, Yoshikazu Inoue, Katsuhisa Uruno; Turkey: Ayse Serdaroglu, Zuhal Yapici, Banu Anlar, Sakir Altunbasak; Russia: Olga Lvova, Oleg Valeryevich Belyaev, Oleg Agranovich, Elena Vladislavovna Levitina, Yulia Vladimirovna Maksimova, Antonina Karas; China: Yuwu Jiang, Liping Zou, Kaifeng Xu, Yushi Zhang, Guoming Luan, Yuqin Zhang, Yi Wang, Meiling Jin, Dingwei Ye, Weiping Liao, Liemin Zhou, Jie Liu, Jianxiang Liao, Bo Yan, Yanchun Deng, Li Jiang, Zhisheng Liu, Shaoping Huang, Hua Li; Korea: Kijoong Kim; Taiwan: Pei-Lung Chen, Hsiu-Fen Lee, Jeng-Dau Tsai, Ching-Shiang Chi, Chao-Ching Huang; Australia: Kate Riney, Deborah Yates, Patrick Kwan; Thailand: Surachai Likasitwattanakul, Charcrin Nabangchang, Lunliya Thampratankul Krisnachai Chomtho, Kamornwan Katanyuwong, Somjit Sriudomkajorn; South Africa: Jo Wilmshurst; Israel: Reeval Segel, Tal Gilboa, Michal Tzadok, Aviva Fattal-Valevski; Greece: Panagiotis Papathanasopoulos, Antigone Syrigou Papavasiliou, Stylianos Giannakodimos, Stylianos Gatzonis, Evangelos Pavlou, Meropi Tzoufi; Netherlands: A. M. H. Vergeer; Belgium: Marc Dhooghe, Hélène Verhelst, Filip Roelens, Marie Cecile Nassogne, Pierre Defresne, Liesbeth De Waele, Patricia Leroy, Nathalie Demonceau, Benjamin Legros, Patrick Van Bogaert, Berten Ceulemans, Lina Dom; France: Pierre Castelnau, Anne De Saint Martin, Audrey Riquet, Mathieu Milh, Claude Cances, Jean-Michel Pedespan, Dorothee Ville, Agathe Roubertie, Stéphane Auvin, Patrick Berquin, Christian Richelme, Catherine Allaire, Sophie Gueden, Sylvie Nguyen The Tich, Bertrand Godet; Spain: Maria Luz Ruiz Falco Rojas, Jaume Campistol Planas, Antonio Martinez Bermejo, Patricia Smeyers Dura, Susana Roldan Aparicio, Maria Jesus Martinez Gonzalez, Javier Lopez Pison, Manuel Oscar Blanco Barca, Eduardo Lopez Laso, Olga Alonso Luengo, Francisco Javier Aguirre Rodriguez, Ignacio Malaga Dieguez, Ana Camacho Salas, Itxaso Marti Carrera, Eduardo Martinez Salcedo, Maria Eugenia Yoldi Petri, Ramon Cancho Candela; Portugal: Ines da Conceicao Carrilho, Jose Pedro Vieira, José Paulo da Silva Oliveira Monteiro, Miguel Jorge Santos de Oliveira Ferreira Leao, Catarina Sofia Marceano Ribeiro Luis, Carla Pires Mendonca; Lithuania: Milda Endziniene; Latvia: Jurgis Strautmanis; Estonia: Inga Talvik; Italy: Maria Paola Canevini, Antonio Gambardella, Dario Pruna, Salvatore Buono, Elena Fontana, Bernardo Dalla Bernardina; Romania: Carmen Burloiu, Iuliu Stefan Bacos Cosma, Mihaela Adela Vintan, Laura Popescu; Czech Republic: Karel Zitterbart; Slovakia: Jaroslava Payerova, Ladislav Bratsky, Zuzana Zilinska; Austria: Ursula Gruber-Sedlmayr, Matthias Baumann, Edda Haberlandt, Kevin Rostasy, Ekaterina Pataraia; United Kingdom: Frances Elmslie, Clare Ann Johnston, Pamela Crawford; Denmark: Peter Uldall; Sweden: Paul Uvebrant, Olof Rask; Norway: Marit Bjoernvold, Eylert Brodtkorb, Andreas Sloerdahl, Ragnar Solhoff, Martine Sofie Gilje Jaatun; Poland: Marek Mandera, Elzbieta Janina Radzikowska, Mariusz Wysocki; Germany: Michael Fischereder, Gerhard Kurlemann, Bernd Wilken, Adelheid Wiemer-Kruel, Klemens Budde, Klaus Marquard, Markus Knuf, Andreas Hahn, Hans Hartmann, Andreas Merkenschlager, Regina Trollmann.

## Conflict of Interest

PV, EB, TC, VC, PC, GB, JK, JF, MF, CF, CH, SJ, RN, FO'C, JQ, MS, RT, MD, JL, AM, SY, MB, BZ, and AJ, received honoraria and support for the travels from Novartis. VC received personal fees for consulting, lecture fees and travel from Actelion, Bayer, Biogen Idec, Boehringer Ingelheim, Gilead, GSK, MSD, Novartis, Pfizer, Roche, Sanofi; grants from Actelion, Boehringer Ingelheim, GSK, Pfizer, Roche; personal fees for developing educational material from Boehringer Ingelheim and Roche. PV has been on the study steering group of the EXIST-1, 2, and 3 studies sponsored by Novartis, and co-PI on two investigator-initiated studies part-funded by Novartis. RN received grant support, paid to her institution, from Eisai and lectures fees from Nutricia, Eisai, Advicenne, and GW Pharma. YT received personal fee from Novartis for lecture and for copyright of referential figures from the journals, and received grant from Japanese government for intractable epilepsy research. SJ was partly financed by the EC Seventh Framework Programme (FP7/2007–2013; EPISTOP, grant agreement no. 602391), the Polish Ministerial funds for science (years 2013–2018) for the implementation of international cofinanced project and the grant EPIMARKER of the Polish National Center for Research and Development No. STRATEGMED3/306306/4/2016. JK, PC, CH, JL, and JQ received research grant from Novartis. RM and SS are employees of Novartis. LD'A was employee of Novartis at the time of manuscript concept approval. The remaining author declares that the research was conducted in the absence of any commercial or financial relationships that could be construed as a potential conflict of interest.
