# Supplementary material for: Tuberous Sclerosis Complex-Associated Neuropsychiatric Disorders (TAND): New Findings on Age, Sex, and Genotype in Relation to Intellectual Phenotype
Source: Front Neurol. 2020 Jul 7;11:603. doi: 10.3389/fneur.2020.00603 (PMC7358578; doi:10.3389/fneur.2020.00603)
Supplement: Supplementary file 1 [file Table_1.docx]

**Supplementary Table 1. TAND features by IQ in Children and Adolescents vs Adults**

|  | | **Children and adolescents (≤18 years)** | | | | **Adults (>18years)** | | | | **Odds ratio**  **(95% CI)** | **P value** |
| --- | --- | --- | --- | --- | --- | --- | --- | --- | --- | --- | --- |
| **TAND manifestation** | | **Total (N=658)**  **N (%)** | **NoID (N=286)**  **N (%)** | **MID (N=202)**  **N (%)** | **M-PID (N=170)**  **N (%)** | **Total (N=236)**  **N (%)** | **NoID (N=109)**  **N (%)** | **MID (N=49)**  **N (%)** | **M-PID (N=78)**  **N (%)** |  |  |
| **Behavioural level** | | | | | | | | | | | |
| Sleep difficulties | Yes* | 120 (40.1) | 27 (28.1) | 37 (37.8) | 56 (53.3) | 52 (40.6) | 19 (39.6) | 8 (25.8) | 25 (51.0) | 0.996 (0.648,1.531) | 0.99 |
|  | No* | 179 (59.9) | 69 (71.9) | 61 (62.2) | 49 (46.7) | 76 (59.4) | 29 (60.4) | 23 (74.2) | 24 (49.0) |  |  |
|  | Total | 299 (45.4) | 96 (33.6) | 98 (48.5) | 105 ( 100) | 128 (54.2) | 48 (44.0) | 31 (63.3) | 49 (62.8) |  |  |
| Severe aggression | Yes* | 65 (21.3) | 15 (15.8) | 26 (25.2) | 24 (22.4) | 35 (28.0) | 7 (15.2) | 11 (33.3) | 17 (37.0) | 0.664 (0.410,1.077) | 0.10 |
|  | No* | 240 (78.7) | 80 (84.2) | 77 (74.8) | 83 (77.6) | 90 (72.0) | 39 (84.8) | 22 (66.7) | 29 (63.0) |  |  |
|  | Total | 305 (46.4) | 95 (33.2) | 103 (51.0) | 107 ( 100) | 125 (53.0) | 46 (42.2) | 33 (67.3) | 46 (59.0) |  |  |
| Self-injury | Yes* | 37 (12.3) | 3 ( 3.2) | 8 ( 8.2) | 26 (24.3) | 26 (20.2) | 5 (11.1) | 6 (17.6) | 15 (30.0) | 0.552 (0.312,0.978) | 0.04* |
|  | No* | 263 (87.7) | 92 (96.8) | 90 (91.8) | 81 (75.7) | 103 (79.8) | 40 (88.9) | 28 (82.4) | 35 (70.0) |  |  |
|  | Total | 300 (45.6) | 95 (33.2) | 98 (48.5) | 107 ( 100) | 129 (54.7) | 45 (41.3) | 34 (69.4) | 50 (64.1) |  |  |
| Impulsivity | Yes* | 151 (50.2) | 40 (42.6) | 58 (57.4) | 53 (50.0) | 50 (40.0) | 17 (37.0) | 12 (38.7) | 21 (43.8) | 1.465 (0.958,2.242) | 0.08 |
|  | No* | 150 (49.8) | 54 (57.4) | 43 (42.6) | 53 (50.0) | 75 (60.0) | 29 (63.0) | 19 (61.3) | 27 (56.3) |  |  |
|  | Total | 301 (45.7) | 94 (32.9) | 101 (50.0) | 106 ( 100) | 125 (53.0) | 46 (42.2) | 31 (63.3) | 48 (61.5) |  |  |
| Overactivity | Yes* | 167 (54.9) | 53 (55.8) | 60 (58.8) | 54 (50.5) | 24 (19.0) | 2 ( 4.3) | 5 (15.6) | 17 (35.4) | 4.944 (3.005,8.134) | <0.0001** |
|  | No* | 137 (45.1) | 42 (44.2) | 42 (41.2) | 53 (49.5) | 102 (81.0) | 44 (95.7) | 27 (84.4) | 31 (64.6) |  |  |
|  | Total | 304 (46.2) | 95 (33.2) | 102 (50.5) | 107 ( 100) | 126 (53.4) | 46 (42.2) | 32 (65.3) | 48 (61.5) |  |  |
| Depression mood | Yes* | 29 ( 9.9) | 12 (12.8) | 9 ( 9.4) | 8 ( 7.8) | 47 (38.2) | 25 (52.1) | 18 (58.1) | 4 ( 9.1) | 0.172 (0.101,0.294) | <0.0001** |
|  | No* | 263 (90.1) | 82 (87.2) | 87 (90.6) | 94 (92.2) | 76 (61.8) | 23 (47.9) | 13 (41.9) | 40 (90.9) |  |  |
|  | Total | 292 (44.4) | 94 (32.9) | 96 (47.5) | 102 ( 100) | 123 (52.1) | 48 (44.0) | 31 (63.3) | 44 (56.4) |  |  |
| Anxiety | Yes* | 81 (27.6) | 24 (25.8) | 34 (34.0) | 23 (23.0) | 65 (52.0) | 32 (68.1) | 20 (58.8) | 13 (29.5) | 0.340 (0.219,0.529) | <0.0001** |
|  | No* | 212 (72.4) | 69 (74.2) | 66 (66.0) | 77 (77.0) | 60 (48.0) | 15 (31.9) | 14 (41.2) | 31 (70.5) |  |  |
|  | Total | 293 (44.5) | 93 (32.5) | 100 (49.5) | 100 ( 100) | 125 (53.0) | 47 (43.1) | 34 (69.4) | 44 (56.4) |  |  |
| Mood swings | Yes* | 82 (28.0) | 17 (18.3) | 37 (37.4) | 28 (27.7) | 52 (42.6) | 19 (43.2) | 13 (44.8) | 20 (40.8) | 0.495 (0.317,0.772) | 0.002** |
|  | No* | 211 (72.0) | 76 (81.7) | 62 (62.6) | 73 (72.3) | 70 (57.4) | 25 (56.8) | 16 (55.2) | 29 (59.2) |  |  |
|  | Total | 293 (44.5) | 93 (32.5) | 99 (49.0) | 101 ( 100) | 122 (51.7) | 44 (40.4) | 29 (59.2) | 49 (62.8) |  |  |
| Obsession | Yes* | 41 (14.1) | 8 ( 8.6) | 15 (15.5) | 18 (17.8) | 30 (24.4) | 2 ( 4.3) | 11 (33.3) | 17 (38.6) | 0.471 (0.275,0.807) | 0.006** |
|  | No* | 250 (85.9) | 85 (91.4) | 82 (84.5) | 83 (82.2) | 93 (75.6) | 44 (95.7) | 22 (66.7) | 27 (61.4) |  |  |
|  | Total | 291 (44.2) | 93 (32.5) | 97 (48.0) | 101 ( 100) | 123 (52.1) | 46 (42.2) | 33 (67.3) | 44 (56.4) |  |  |
| Hallucination | Yes* | 2 ( 0.7) | 1 ( 1.1) | 1 ( 1.1) | 0 | 16 (12.5) | 4 ( 8.3) | 8 (23.5) | 4 ( 8.7) | 0.046 (0.010,0.204) | <0.0001** |
|  | No* | 286 (99.3) | 94 (98.9) | 94 (98.9) | 98 ( 100) | 112 (87.5) | 44 (91.7) | 26 (76.5) | 42 (91.3) |  |  |
|  | Total | 288 (43.8) | 95 (33.2) | 95 (47.0) | 98 ( 100) | 128 (54.2) | 48 (44.0) | 34 (69.4) | 46 (59.0) |  |  |
| Psychosis | Yes* | 9 ( 3.1) | 2 ( 2.0) | 7 ( 7.0) | 0 | 16 (12.7) | 3 ( 6.3) | 9 (26.5) | 4 ( 9.1) | 0.209 (0.090,0.486) | <0.0001** |
|  | No* | 283 (96.9) | 94 ( 100) | 96 (98.0) | 93 (93.0) | 110 (87.3) | 45 (93.8) | 25 (73.5) | 40 (90.9) |  |  |
|  | Total | 292 (44.4) | 94 (32.9) | 98 (48.5) | 100 ( 100) | 126 (53.4) | 48 (44.0) | 34 (69.4) | 44 (56.4) |  |  |
| **Psychiatric level** | | | | | | | | | | | |
| Autism spectrum disorder | Yes* | 127 (21.7) | 11 ( 4.2) | 27 (15.6) | 89 (58.2) | 38 (19.0) | 3 ( 3.3) | 4 ( 8.9) | 31 (49.2) | 1.505 (0.930,2.435) | 0.10 |
|  | No* | 459 (78.3) | 249 (95.8) | 146 (84.4) | 64 (41.8) | 162 (81.0) | 89 (96.7) | 41 (91.1) | 32 (50.8) |  |  |
|  | Total | 586 (89.1) | 260 (90.9) | 173 (85.6) | 153 ( 100) | 200 (84.7) | 92 (84.4) | 45 (91.8) | 63 (80.8) |  |  |
| Attention deficit hyperactivity disorder | Yes* | 144 (25.2) | 51 (19.6) | 51 (29.5) | 42 (30.4) | 23 (12.7) | 5 ( 5.6) | 4 ( 9.3) | 14 (28.6) | 2.359 (1.450,3.837) | 0.0004** |
|  | No* | 427 (74.8) | 209 (80.4) | 122 (70.5) | 96 (69.6) | 158 (87.3) | 84 (94.4) | 39 (90.7) | 35 (71.4) |  |  |
|  | Total | 571 (86.8) | 260 (90.9) | 173 (85.6) | 138 ( 100) | 181 (76.7) | 89 (81.7) | 43 (87.8) | 49 (62.8) |  |  |
| Depressive disorder | Yes* | 13 ( 2.3) | 7 ( 2.7) | 2 ( 1.2) | 4 ( 2.9) | 29 (16.1) | 16 (18.6) | 11 (26.8) | 2 ( 3.8) | 0.128 (0.066,0.248) | <0.0001** |
|  | No* | 549 (97.7) | 252 (97.3) | 165 (98.8) | 132 (97.1) | 151 (83.9) | 70 (81.4) | 30 (73.2) | 51 (96.2) |  |  |
|  | Total | 562 (85.4) | 259 (90.6) | 167 (82.7) | 136 ( 100) | 180 (76.3) | 86 (78.9) | 41 (83.7) | 53 (67.9) |  |  |
| Anxiety disorder | Yes* | 51 ( 9.1) | 20 ( 7.7) | 17 (10.2) | 14 (10.4) | 36 (19.7) | 18 (20.7) | 11 (26.2) | 7 (13.0) | 0.403 (0.253,0.642) | <0.0001** |
|  | No* | 509 (90.9) | 239 (92.3) | 149 (89.8) | 121 (89.6) | 147 (80.3) | 69 (79.3) | 31 (73.8) | 47 (87.0) |  |  |
|  | Total | 560 (85.1) | 259 (90.6) | 166 (82.2) | 135 ( 100) | 183 (77.5) | 87 (79.8) | 42 (85.7) | 54 (69.2) |  |  |
| Other psychiatric disorder | Yes* | 32 ( 5.7) | 8 ( 3.1) | 11 ( 6.6) | 13 ( 9.4) | 29 (15.7) | 9 (10.0) | 9 (21.4) | 11 (20.8) | 0.317 (0.185,0.544) | <0.0001** |
|  | No* | 531 (94.3) | 251 (96.9) | 155 (93.4) | 125 (90.6) | 156 (84.3) | 81 (90.0) | 33 (78.6) | 42 (79.2) |  |  |
|  | Total | 563 (85.6) | 259 (90.6) | 166 (82.2) | 138 ( 100) | 185 (78.4) | 90 (82.6) | 42 (85.7) | 53 (67.9) |  |  |
| **Academic level** | | | | | | | | | | | |
| Patients with academy/scholastic skills difficulties | Yes* | 322 (68.5) | 100 (48.5) | 121 (80.1) | 101 (89.4) | 128 (66.7) | 43 (44.3) | 35 (92.1) | 50 (87.7) | 0.991 (0.664,1.478) | 0.96 |
|  | No* | 148 (31.5) | 106 (51.5) | 30 (19.9) | 12 (10.6) | 64 (33.3) | 54 (55.7) | 3 ( 7.9) | 7 (12.3) |  |  |
|  | Total | 470 (71.4) | 206 (72.0) | 151 (74.8) | 113 ( 100) | 192 (81.4) | 97 (89.0) | 38 (77.6) | 57 (73.1) |  |  |
| Patients with assessed difficulties | Yes* | 219 (79.3) | 77 (84.6) | 82 (80.4) | 60 (72.3) | 71 (70.3) | 19 (51.4) | 21 (77.8) | 31 (83.8) | 1.565 (0.940,2.604) | 0.08 |
|  | No* | 57 (20.7) | 14 (15.4) | 20 (19.6) | 23 (27.7) | 30 (29.7) | 18 (48.6) | 6 (22.2) | 6 (16.2) |  |  |
|  | Total | 276 (41.9) | 91 (31.8) | 102 (50.5) | 83 ( 100) | 101 (42.8) | 37 (33.9) | 27 (55.1) | 37 (47.4) |  |  |
| **Neuro-psychological level** | | | | | | | | | | | |
| Patients with neuropsychological skills assessed | Yes* | 297 (57.4) | 130 (56.3) | 99 (60.0) | 68 (56.2) | 111 (60.0) | 53 (57.0) | 24 (64.9) | 34 (61.8) | 0.882 (0.625,1.246) | 0.48 |
|  | No* | 220 (42.6) | 101 (43.7) | 66 (40.0) | 53 (43.8) | 74 (40.0) | 40 (43.0) | 13 (35.1) | 21 (38.2) |  |  |
|  | Total | 517 (78.6) | 231 (80.8) | 165 (81.7) | 121 ( 100) | 185 (78.4) | 93 (85.3) | 37 (75.5) | 55 (70.5) |  |  |
| Patients with any deficit (Performance<5th percentile) | Yes* | 181 (70.2) | 50 (42.7) | 74 (89.2) | 57 (98.3) | 69 (68.3) | 19 (38.0) | 18 (94.7) | 32 ( 100) | 1.046 (0.559,1.957) | 0.89 |
|  | No* | 77 (29.8) | 67 (57.3) | 9 (10.8) | 1 ( 1.7) | 32 (31.7) | 31 (62.0) | 1 ( 5.3) | 0 |  |  |
|  | Total | 258 (39.2) | 117 (40.9) | 83 (41.1) | 58 ( 100) | 101 (42.8) | 50 (45.9) | 19 (38.8) | 32 (41.0) |  |  |

NoID: Normal (IQ >70); MID: Mild intellectual disability (IQ 51-70) M-PID: Moderate to profound intellectual disability (IQ 36-< 20)

*Percentages are calculated from the total number of patients with yes and no answers.

#P value calculated from chi-square to test association between categories of intellectual disability (NoID, MID and M-PID) and presence of respective TAND manifestation.
