# Supplementary material for: Tuberous Sclerosis Complex-Associated Neuropsychiatric Disorders (TAND): New Findings on Age, Sex, and Genotype in Relation to Intellectual Phenotype
Source: Front Neurol. 2020 Jul 7;11:603. doi: 10.3389/fneur.2020.00603 (PMC7358578; doi:10.3389/fneur.2020.00603)
Supplement: Supplementary file 2 [file Table_2.docx]

**Supplementary Table 2. TAND features by IQ in Male vs Female**

|  | | **Male** | | | | **Female** | | | | **Odds ratio**  **(95% CI)** | **P value** |
| --- | --- | --- | --- | --- | --- | --- | --- | --- | --- | --- | --- |
| **TAND manifestation** | | **Total (N=432)**  **N (%)** | **NoID (N=176)**  **N (%)** | **MID (N=123)**  **N (%)** | **M-PID (N=133)**  **N (%)** | **Total (N=462)**  **N (%)** | **NoID (N=219)**  **N (%)** | **MID (N=128)**  **N (%)** | **M-PID (N=115)**  **N (%)** |  |  |
| **Behavioural level** | | | | | | | | | | | |
| Sleep difficulties | Yes* | 92 (42.0) | 18 (26.1) | 24 (35.3) | 50 (61.0) | 80 (38.5) | 28 (37.3) | 21 (34.4) | 31 (43.1) | 0.883 (0.597,1.307) | 0.53 |
|  | No* | 127 (58.0) | 51 (73.9) | 44 (64.7) | 32 (39.0) | 128 (61.5) | 47 (62.7) | 40 (65.6) | 41 (56.9) |  |  |
|  | Total | 219 (50.7) | 69 (39.2) | 68 (55.3) | 82 (61.7) | 208 (45.0) | 75 (34.2) | 61 (47.7) | 72 (62.6) |  |  |
| Severe aggression | Yes* | 54 (24.5) | 14 (20.6) | 21 (29.2) | 19 (23.8) | 46 (21.9) | 8 (11.0) | 16 (25.0) | 22 (30.1) | 0.883 (0.563,1.385) | 0.59 |
|  | No* | 166 (75.5) | 54 (79.4) | 51 (70.8) | 61 (76.3) | 164 (78.1) | 65 (89.0) | 48 (75.0) | 51 (69.9) |  |  |
|  | Total | 220 (50.9) | 68 (38.6) | 72 (58.5) | 80 (60.2) | 210 (45.5) | 73 (33.3) | 64 (50.0) | 73 (63.5) |  |  |
| Self-injury | Yes* | 34 (15.3) | 4 ( 6.0) | 6 ( 8.3) | 24 (28.9) | 29 (14.0) | 4 ( 5.5) | 8 (13.3) | 17 (23.0) | 0.934 (0.538,1.622) | 0.81 |
|  | No* | 188 (84.7) | 63 (94.0) | 66 (91.7) | 59 (71.1) | 178 (86.0) | 69 (94.5) | 52 (86.7) | 57 (77.0) |  |  |
|  | Total | 222 (51.4) | 67 (38.1) | 72 (58.5) | 83 (62.4) | 207 (44.8) | 73 (33.3) | 60 (46.9) | 74 (64.3) |  |  |
| Impulsivity | Yes* | 117 (53.4) | 32 (47.8) | 45 (64.3) | 40 (48.8) | 84 (40.6) | 25 (34.2) | 25 (40.3) | 34 (47.2) | 0.606 (0.412,0.889) | 0.01* |
|  | No* | 102 (46.6) | 35 (52.2) | 25 (35.7) | 42 (51.2) | 123 (59.4) | 48 (65.8) | 37 (59.7) | 38 (52.8) |  |  |
|  | Total | 219 (50.7) | 67 (38.1) | 70 (56.9) | 82 (61.7) | 207 (44.8) | 73 (33.3) | 62 (48.4) | 72 (62.6) |  |  |
| Overactivity | Yes* | 124 (55.6) | 33 (49.3) | 46 (63.0) | 45 (54.2) | 67 (32.4) | 22 (29.7) | 19 (31.1) | 26 (36.1) | 0.388 (0.262,0.575) | <0.0001** |
|  | No* | 99 (44.4) | 34 (50.7) | 27 (37.0) | 38 (45.8) | 140 (67.6) | 52 (70.3) | 42 (68.9) | 46 (63.9) |  |  |
|  | Total | 223 (51.6) | 67 (38.1) | 73 (59.3) | 83 (62.4) | 207 (44.8) | 74 (33.8) | 61 (47.7) | 72 (62.6) |  |  |
| Depression mood | Yes* | 34 (15.9) | 16 (23.9) | 11 (15.9) | 7 ( 9.0) | 42 (20.9) | 21 (28.0) | 16 (27.6) | 5 ( 7.4) | 1.356 (0.815,2.256) | 0.24 |
|  | No* | 180 (84.1) | 51 (76.1) | 58 (84.1) | 71 (91.0) | 159 (79.1) | 54 (72.0) | 42 (72.4) | 63 (92.6) |  |  |
|  | Total | 214 (49.5) | 67 (38.1) | 69 (56.1) | 78 (58.6) | 201 (43.5) | 75 (34.2) | 58 (45.3) | 68 (59.1) |  |  |
| Anxiety | Yes* | 62 (28.8) | 23 (34.8) | 22 (30.6) | 17 (22.1) | 84 (41.4) | 33 (44.6) | 32 (51.6) | 19 (28.4) | 1.731 (1.148,2.609) | 0.009** |
|  | No* | 153 (71.2) | 43 (65.2) | 50 (69.4) | 60 (77.9) | 119 (58.6) | 41 (55.4) | 30 (48.4) | 48 (71.6) |  |  |
|  | Total | 215 (49.8) | 66 (37.5) | 72 (58.5) | 77 (57.9) | 203 (43.9) | 74 (33.8) | 62 (48.4) | 67 (58.3) |  |  |
| Mood swings | Yes* | 62 (28.8) | 14 (21.9) | 21 (30.0) | 27 (33.3) | 72 (36.0) | 22 (30.1) | 29 (50.0) | 21 (30.4) | 1.434 (0.947,2.172) | 0.09 |
|  | No* | 153 (71.2) | 50 (78.1) | 49 (70.0) | 54 (66.7) | 128 (64.0) | 51 (69.9) | 29 (50.0) | 48 (69.6) |  |  |
|  | Total | 215 (49.8) | 64 (36.4) | 70 (56.9) | 81 (60.9) | 200 (43.3) | 73 (33.3) | 58 (45.3) | 69 (60.0) |  |  |
| Obsession | Yes* | 45 (21.0) | 5 ( 7.7) | 16 (22.2) | 24 (31.2) | 26 (13.0) | 5 ( 6.8) | 10 (17.2) | 11 (16.2) | 0.588 (0.344,1.005) | 0.05 |
|  | No* | 169 (79.0) | 60 (92.3) | 56 (77.8) | 53 (68.8) | 174 (87.0) | 69 (93.2) | 48 (82.8) | 57 (83.8) |  |  |
|  | Total | 214 (49.5) | 65 (36.9) | 72 (58.5) | 77 (57.9) | 200 (43.3) | 74 (33.8) | 58 (45.3) | 68 (59.1) |  |  |
| Hallucination | Yes* | 6 ( 2.8) | 2 ( 2.9) | 3 ( 4.2) | 1 ( 1.3) | 12 ( 5.9) | 3 ( 4.0) | 6 (10.3) | 3 ( 4.3) | 2.277 (0.836,6.198) | 0.10 |
|  | No* | 208 (97.2) | 66 (97.1) | 68 (95.8) | 74 (98.7) | 190 (94.1) | 72 (96.0) | 52 (89.7) | 66 (95.7) |  |  |
|  | Total | 214 (49.5) | 68 (38.6) | 71 (57.7) | 75 (56.4) | 202 (43.7) | 75 (34.2) | 58 (45.3) | 69 (60.0) |  |  |
| Psychosis | Yes* | 13 ( 6.0) | 2 ( 3.0) | 4 ( 5.6) | 7 ( 9.2) | 12 ( 5.9) | 1 ( 1.3) | 7 (11.7) | 4 ( 5.9) | 1.038 (0.463,2.331) | 0.93 |
|  | No* | 202 (94.0) | 65 (97.0) | 68 (94.4) | 69 (90.8) | 191 (94.1) | 74 (98.7) | 53 (88.3) | 64 (94.1) |  |  |
|  | Total | 215 (49.8) | 67 (38.1) | 72 (58.5) | 76 (57.1) | 203 (43.9) | 75 (34.2) | 60 (46.9) | 68 (59.1) |  |  |
| **Psychiatric level** | | | | | | | | | | | |
| Autism spectrum disorder | Yes* | 110 (28.9) | 8 ( 5.2) | 22 (20.2) | 80 (67.8) | 55 (13.5) | 6 ( 3.0) | 9 ( 8.3) | 40 (40.8) | 0.364 (0.238,0.557) | <0.0001** |
|  | No* | 270 (71.1) | 145 (94.8) | 87 (79.8) | 38 (32.2) | 351 (86.5) | 193 (97.0) | 100 (91.7) | 58 (59.2) |  |  |
|  | Total | 380 (88.0) | 153 (86.9) | 109 (88.6) | 118 (88.7) | 406 (87.9) | 199 (90.9) | 109 (85.2) | 98 (85.2) |  |  |
| Attention deficit hyperactivity disorder | Yes* | 102 (28.1) | 29 (19.0) | 38 (36.2) | 35 (33.3) | 65 (16.7) | 27 (13.8) | 17 (15.3) | 21 (25.6) | 0.541 (0.380,0.771) | 0.0006** |
|  | No* | 261 (71.9) | 124 (81.0) | 67 (63.8) | 70 (66.7) | 324 (83.3) | 169 (86.2) | 94 (84.7) | 61 (74.4) |  |  |
|  | Total | 363 (84.0) | 153 (86.9) | 105 (85.4) | 105 (78.9) | 389 (84.2) | 196 (89.5) | 111 (86.7) | 82 (71.3) |  |  |
| Depressive disorder | Yes* | 17 ( 4.8) | 10 ( 6.7) | 5 ( 5.0) | 2 ( 2.0) | 25 ( 6.4) | 13 ( 6.7) | 8 ( 7.5) | 4 ( 4.6) | 1.299 (0.690,2.446) | 0.42 |
|  | No* | 336 (95.2) | 140 (93.3) | 96 (95.0) | 100 (98.0) | 364 (93.6) | 182 (93.3) | 99 (92.5) | 83 (95.4) |  |  |
|  | Total | 353 (81.7) | 150 (85.2) | 101 (82.1) | 102 (76.7) | 389 (84.2) | 195 (89.0) | 107 (83.6) | 87 (75.7) |  |  |
| Anxiety disorder | Yes* | 38 (10.7) | 17 (11.3) | 12 (11.7) | 9 ( 8.9) | 49 (12.6) | 21 (10.8) | 16 (15.2) | 12 (13.6) | 1.212 (0.773,1.902) | 0.40 |
|  | No* | 317 (89.3) | 134 (88.7) | 91 (88.3) | 92 (91.1) | 339 (87.4) | 174 (89.2) | 89 (84.8) | 76 (86.4) |  |  |
|  | Total | 355 (82.2) | 151 (85.8) | 103 (83.7) | 101 (75.9) | 388 (84.0) | 195 (89.0) | 105 (82.0) | 88 (76.5) |  |  |
| Other psychiatric disorder | Yes* | 25 ( 7.1) | 7 ( 4.7) | 8 ( 7.8) | 10 ( 9.9) | 36 ( 9.1) | 10 ( 5.0) | 12 (11.3) | 14 (15.6) | 1.422 (0.832,2.430) | 0.20 |
|  | No* | 328 (92.9) | 143 (95.3) | 94 (92.2) | 91 (90.1) | 359 (90.9) | 189 (95.0) | 94 (88.7) | 76 (84.4) |  |  |
|  | Total | 353 (81.7) | 150 (85.2) | 102 (82.9) | 101 (75.9) | 395 (85.5) | 199 (90.9) | 106 (82.8) | 90 (78.3) |  |  |
| **Academic level** | | | | | | | | | | | |
| Patients with academy/scholastic skills difficulties | Yes* | 211 (68.7) | 59 (46.8) | 73 (80.2) | 79 (87.8) | 239 (67.3) | 84 (47.5) | 83 (84.7) | 72 (90.0) | 1.127 (0.785,1.619) | 0.52 |
|  | No* | 96 (31.3) | 67 (53.2) | 18 (19.8) | 11 (12.2) | 116 (32.7) | 93 (52.5) | 15 (15.3) | 8 (10.0) |  |  |
|  | Total | 307 (71.1) | 126 (71.6) | 91 (74.0) | 90 (67.7) | 355 (76.8) | 177 (80.8) | 98 (76.6) | 80 (69.6) |  |  |
| Patients with assessed difficulties | Yes* | 146 (82.0) | 42 (79.2) | 52 (81.3) | 52 (85.2) | 144 (72.4) | 54 (72.0) | 51 (78.5) | 39 (66.1) | 0.581 (0.355,0.950) | 0.03 * |
|  | No* | 32 (18.0) | 11 (20.8) | 12 (18.8) | 9 (14.8) | 55 (27.6) | 21 (28.0) | 14 (21.5) | 20 (33.9) |  |  |
|  | Total | 178 (41.2) | 53 (30.1) | 64 (52.0) | 61 (45.9) | 199 (43.1) | 75 (34.2) | 65 (50.8) | 59 (51.3) |  |  |
| **Neuro-psychological level** | | | | | | | | | | | |
| Patients with neuropsychological skills assessed | Yes* | 194 (57.4) | 77 (55.4) | 63 (60.6) | 54 (56.8) | 214 (58.8) | 106 (57.3) | 60 (61.2) | 48 (59.3) | 1.071 (0.792,1.448) | 0.66 |
|  | No* | 144 (42.6) | 62 (44.6) | 41 (39.4) | 41 (43.2) | 150 (41.2) | 79 (42.7) | 38 (38.8) | 33 (40.7) |  |  |
|  | Total | 338 (78.2) | 139 (79.0) | 104 (84.6) | 95 (71.4) | 364 (78.8) | 185 (84.5) | 98 (76.6) | 81 (70.4) |  |  |
| Patients with any deficit (Performance<5th percentile) | Yes* | 126 (74.6) | 33 (46.5) | 46 (92.0) | 47 (97.9) | 124 (65.3) | 36 (37.5) | 46 (88.5) | 42 ( 100) | 0.718 (0.412,1.254) | 0.25 |
|  | No* | 43 (25.4) | 38 (53.5) | 4 ( 8.0) | 1 ( 2.1) | 66 (34.7) | 60 (62.5) | 6 (11.5) | 0 |  |  |
|  | Total | 169 (39.1) | 71 (40.3) | 50 (40.7) | 48 (36.1) | 190 (41.1) | 96 (43.8) | 52 (40.6) | 42 (36.5) |  |  |

NoID: Normal (IQ >70); MID: Mild intellectual disability (IQ 51-70) M-PID: Moderate to profound intellectual disability (IQ 36-< 20)

*Percentages are calculated from the total number of patients with yes and no answers.

#P value calculated from chi-square to test association between categories of intellectual disability (NoID, MID and M-PID) and presence of respective TAND manifestation.
