# Supplementary material for: Tuberous Sclerosis Complex-Associated Neuropsychiatric Disorders (TAND): New Findings on Age, Sex, and Genotype in Relation to Intellectual Phenotype
Source: Front Neurol. 2020 Jul 7;11:603. doi: 10.3389/fneur.2020.00603 (PMC7358578; doi:10.3389/fneur.2020.00603)
Supplement: Supplementary file 3 [file Table_3.docx]

**Supplementary Table 3. TAND features by IQ in *TSC1* vs *TSC2***

|  | | ***TSC1*** | | | | ***TSC2*** | | | | **Odds ratio**  **(95% CI)** | **P value** |
| --- | --- | --- | --- | --- | --- | --- | --- | --- | --- | --- | --- |
| **TAND manifestation** | | **Total (N=94)**  **N (%)** | **NoID (N=61)**  **N (%)** | **MID (N=16)**  **N (%)** | **M-PID (N=17)**  **N (%)** | **Total (N=301)**  **N (%)** | **NoID (N=126)**  **N (%)** | **MID (N=76)**  **N (%)** | **M-PID (N=99)**  **N (%)** |  |  |
| **Behavioural level** | | | | | | | | | | | |
| Sleep difficulties | Yes* | 12 (31.6) | 5 (25.0) | 1 (14.3) | 6 (54.5) | 66 (45.2) | 16 (35.6) | 17 (41.5) | 33 (55.0) | 1.624 (0.739,3.568) | 0.23 |
|  | No* | 26 (68.4) | 15 (75.0) | 6 (85.7) | 5 (45.5) | 80 (54.8) | 29 (64.4) | 24 (58.5) | 27 (45.0) |  |  |
|  | Total | 38 (40.4) | 20 (32.8) | 7 (43.8) | 11 (64.7) | 146 (48.5) | 45 (35.7) | 41 (53.9) | 60 (60.6) |  |  |
| Severe aggression | Yes* | 11 (27.5) | 4 (20.0) | 3 (33.3) | 4 (36.4) | 27 (18.0) | 7 (15.2) | 9 (20.9) | 11 (18.0) | 0.536 (0.235,1.223) | 0.14 |
|  | No* | 29 (72.5) | 16 (80.0) | 6 (66.7) | 7 (63.6) | 123 (82.0) | 39 (84.8) | 34 (79.1) | 50 (82.0) |  |  |
|  | Total | 40 (42.6) | 20 (32.8) | 9 (56.3) | 11 (64.7) | 150 (49.8) | 46 (36.5) | 43 (56.6) | 61 (61.6) |  |  |
| Self-injury | Yes* | 1 ( 2.6) | 0 | 0 | 1 ( 9.1) | 24 (16.1) | 4 ( 8.9) | 7 (16.7) | 13 (21.0) | 6.155 (0.796,47.597) | 0.05 * |
|  | No* | 38 (97.4) | 20 ( 100) | 8 ( 100) | 10 (90.9) | 125 (83.9) | 41 (91.1) | 35 (83.3) | 49 (79.0) |  |  |
|  | Total | 39 (41.5) | 20 (32.8) | 8 (50.0) | 11 (64.7) | 149 (49.5) | 45 (35.7) | 42 (55.3) | 62 (62.6) |  |  |
| Impulsivity | Yes* | 23 (59.0) | 11 (52.4) | 4 (57.1) | 8 (72.7) | 68 (45.6) | 19 (41.3) | 25 (59.5) | 24 (39.3) | 0.540 (0.260,1.121) | 0.09 |
|  | No* | 16 (41.0) | 10 (47.6) | 3 (42.9) | 3 (27.3) | 81 (54.4) | 27 (58.7) | 17 (40.5) | 37 (60.7) |  |  |
|  | Total | 39 (41.5) | 21 (34.4) | 7 (43.8) | 11 (64.7) | 149 (49.5) | 46 (36.5) | 42 (55.3) | 61 (61.6) |  |  |
| Overactivity | Yes* | 18 (45.0) | 8 (40.0) | 5 (55.6) | 5 (45.5) | 61 (41.2) | 14 (31.1) | 19 (46.3) | 28 (45.2) | 0.768 (0.373,1.580) | 0.48 |
|  | No* | 22 (55.0) | 12 (60.0) | 4 (44.4) | 6 (54.5) | 87 (58.8) | 31 (68.9) | 22 (53.7) | 34 (54.8) |  |  |
|  | Total | 40 (42.6) | 20 (32.8) | 9 (56.3) | 11 (64.7) | 148 (49.2) | 45 (35.7) | 41 (53.9) | 62 (62.6) |  |  |
| Depression mood | Yes* | 10 (27.0) | 6 (30.0) | 3 (42.9) | 1 (10.0) | 25 (17.7) | 10 (22.2) | 10 (25.0) | 5 ( 8.9) | 0.623 (0.258,1.504) | 0.29 |
|  | No* | 27 (73.0) | 14 (70.0) | 4 (57.1) | 9 (90.0) | 116 (82.3) | 35 (77.8) | 30 (75.0) | 51 (91.1) |  |  |
|  | Total | 37 (39.4) | 20 (32.8) | 7 (43.8) | 10 (58.8) | 141 (46.8) | 45 (35.7) | 40 (52.6) | 56 (56.6) |  |  |
| Anxiety | Yes* | 14 (37.8) | 8 (40.0) | 4 (50.0) | 2 (22.2) | 56 (38.9) | 20 (44.4) | 19 (45.2) | 17 (29.8) | 1.145 (0.531,2.469) | 0.73 |
|  | No* | 23 (62.2) | 12 (60.0) | 4 (50.0) | 7 (77.8) | 88 (61.1) | 25 (55.6) | 23 (54.8) | 40 (70.2) |  |  |
|  | Total | 37 (39.4) | 20 (32.8) | 8 (50.0) | 9 (52.9) | 144 (47.8) | 45 (35.7) | 42 (55.3) | 57 (57.6) |  |  |
| Mood swings | Yes* | 13 (32.5) | 5 (23.8) | 5 (55.6) | 3 (30.0) | 39 (27.9) | 9 (20.9) | 17 (42.5) | 13 (22.8) | 0.716 (0.323,1.588) | 0.41 |
|  | No* | 27 (67.5) | 16 (76.2) | 4 (44.4) | 7 (70.0) | 101 (72.1) | 34 (79.1) | 23 (57.5) | 44 (77.2) |  |  |
|  | Total | 40 (42.6) | 21 (34.4) | 9 (56.3) | 10 (58.8) | 140 (46.5) | 43 (34.1) | 40 (52.6) | 57 (57.6) |  |  |
| Obsession | Yes* | 6 (16.2) | 2 (10.0) | 2 (25.0) | 2 (22.2) | 24 (16.8) | 3 ( 6.8) | 6 (14.6) | 15 (25.9) | 0.788 (0.284,2.185) | 0.65 |
|  | No* | 31 (83.8) | 18 (90.0) | 6 (75.0) | 7 (77.8) | 119 (83.2) | 41 (93.2) | 35 (85.4) | 43 (74.1) |  |  |
|  | Total | 37 (39.4) | 20 (32.8) | 8 (50.0) | 9 (52.9) | 143 (47.5) | 44 (34.9) | 41 (53.9) | 58 (58.6) |  |  |
| Hallucination | Yes* | 2 ( 5.3) | 1 ( 5.0) | 1 (12.5) | 0 | 6 ( 4.2) | 2 ( 4.3) | 2 ( 5.0) | 2 ( 3.4) | 0.796 (0.149,4.247) | 0.79 |
|  | No* | 36 (94.7) | 19 (95.0) | 7 (87.5) | 10 ( 100) | 138 (95.8) | 44 (95.7) | 38 (95.0) | 56 (96.6) |  |  |
|  | Total | 38 (40.4) | 20 (32.8) | 8 (50.0) | 10 (58.8) | 144 (47.8) | 46 (36.5) | 40 (52.6) | 58 (58.6) |  |  |
| Psychosis | Yes* | 3 ( 7.9) | 1 ( 5.0) | 2 (25.0) | 0 | 5 ( 3.5) | 0 | 2 ( 4.8) | 3 ( 5.3) | 0.300 (0.063,1.423) | 0.12 |
|  | No* | 35 (92.1) | 19 (95.0) | 6 (75.0) | 10 ( 100) | 139 (96.5) | 45 ( 100) | 40 (95.2) | 54 (94.7) |  |  |
|  | Total | 38 (40.4) | 20 (32.8) | 8 (50.0) | 10 (58.8) | 144 (47.8) | 45 (35.7) | 42 (55.3) | 57 (57.6) |  |  |
| **Psychiatric level** | | | | | | | | | | | |
| Autism spectrum disorder | Yes* | 8 ( 9.4) | 2 ( 3.5) | 2 (13.3) | 4 (30.8) | 73 (26.8) | 5 ( 4.4) | 10 (15.6) | 58 (61.1) | 2.020 (0.857,4.761) | 0.09 |
|  | No* | 77 (90.6) | 55 (96.5) | 13 (86.7) | 9 (69.2) | 199 (73.2) | 108 (95.6) | 54 (84.4) | 37 (38.9) |  |  |
|  | Total | 85 (90.4) | 57 (93.4) | 15 (93.8) | 13 (76.5) | 272 (90.4) | 113 (89.7) | 64 (84.2) | 95 (96.0) |  |  |
| Attention deficit hyperactivity disorder | Yes* | 18 (22.2) | 8 (14.0) | 4 (30.8) | 6 (54.5) | 46 (18.0) | 13 (11.4) | 13 (20.0) | 20 (26.0) | 0.564 (0.294,1.083) | 0.08 |
|  | No* | 63 (77.8) | 49 (86.0) | 9 (69.2) | 5 (45.5) | 210 (82.0) | 101 (88.6) | 52 (80.0) | 57 (74.0) |  |  |
|  | Total | 81 (86.2) | 57 (93.4) | 13 (81.3) | 11 (64.7) | 256 (85.0) | 114 (90.5) | 65 (85.5) | 77 (77.8) |  |  |
| Depressive disorder | Yes* | 8 ( 9.9) | 5 ( 8.9) | 3 (23.1) | 0 | 12 ( 4.7) | 5 ( 4.5) | 5 ( 7.8) | 2 ( 2.5) | 0.443 (0.168,1.170) | 0.09 |
|  | No* | 73 (90.1) | 51 (91.1) | 10 (76.9) | 12 ( 100) | 244 (95.3) | 107 (95.5) | 59 (92.2) | 78 (97.5) |  |  |
|  | Total | 81 (86.2) | 56 (91.8) | 13 (81.3) | 12 (70.6) | 256 (85.0) | 112 (88.9) | 64 (84.2) | 80 (80.8) |  |  |
| Anxiety disorder | Yes* | 9 (11.1) | 5 ( 8.9) | 3 (23.1) | 1 ( 8.3) | 26 (10.2) | 13 (11.6) | 7 (11.3) | 6 ( 7.5) | 0.941 (0.418,2.117) | 0.88 |
|  | No* | 72 (88.9) | 51 (91.1) | 10 (76.9) | 11 (91.7) | 228 (89.8) | 99 (88.4) | 55 (88.7) | 74 (92.5) |  |  |
|  | Total | 81 (86.2) | 56 (91.8) | 13 (81.3) | 12 (70.6) | 254 (84.4) | 112 (88.9) | 62 (81.6) | 80 (80.8) |  |  |
| Other psychiatric disorder | Yes* | 6 ( 7.4) | 3 ( 5.2) | 1 ( 9.1) | 2 (16.7) | 16 ( 6.3) | 5 ( 4.5) | 6 ( 9.8) | 5 ( 6.0) | 0.700 (0.257,1.904) | 0.48 |
|  | No* | 75 (92.6) | 55 (94.8) | 10 (90.9) | 10 (83.3) | 238 (93.7) | 105 (95.5) | 55 (90.2) | 78 (94.0) |  |  |
|  | Total | 81 (86.2) | 58 (95.1) | 11 (68.8) | 12 (70.6) | 254 (84.4) | 110 (87.3) | 61 (80.3) | 83 (83.8) |  |  |
| **Academic level** | | | | | | | | | | | |
| Patients with academy/scholastic skills difficulties | Yes* | 46 (62.2) | 22 (45.8) | 13 ( 100) | 11 (84.6) | 163 (72.8) | 51 (51.5) | 49 (83.1) | 63 (95.5) | 1.128 (0.608,2.094) | 0.70 |
|  | No* | 28 (37.8) | 26 (54.2) |  | 2 (15.4) | 61 (27.2) | 48 (48.5) | 10 (16.9) | 3 ( 4.5) |  |  |
|  | Total | 74 (78.7) | 48 (78.7) | 13 (81.3) | 13 (76.5) | 224 (74.4) | 99 (78.6) | 59 (77.6) | 66 (66.7) |  |  |
| Patients with assessed difficulties | Yes* | 31 (79.5) | 15 (75.0) | 9 (90.0) | 7 (77.8) | 108 (77.1) | 35 (72.9) | 32 (76.2) | 41 (82.0) | 0.808 (0.334,1.954) | 0.64 |
|  | No* | 8 (20.5) | 5 (25.0) | 1 (10.0) | 2 (22.2) | 32 (22.9) | 13 (27.1) | 10 (23.8) | 9 (18.0) |  |  |
|  | Total | 39 (41.5) | 20 (32.8) | 10 (62.5) | 9 (52.9) | 140 (46.5) | 48 (38.1) | 42 (55.3) | 50 (50.5) |  |  |
| **Neuro-psychological level** | | | | | | | | | | | |
| Patients with neuropsychological skills assessed | Yes* | 51 (67.1) | 35 (70.0) | 10 (83.3) | 6 (42.9) | 160 (65.6) | 68 (63.0) | 41 (66.1) | 51 (68.9) | 0.920 (0.530,1.596) | 0.76 |
|  | No* | 25 (32.9) | 15 (30.0) | 2 (16.7) | 8 (57.1) | 84 (34.4) | 40 (37.0) | 21 (33.9) | 23 (31.1) |  |  |
|  | Total | 76 (80.9) | 50 (82.0) | 12 (75.0) | 14 (82.4) | 244 (81.1) | 108 (85.7) | 62 (81.6) | 74 (74.7) |  |  |
| Patients with any deficit (Performance<5th percentile) | Yes* | 24 (54.5) | 13 (40.6) | 7 ( 100) | 4 (80.0) | 112 (76.7) | 30 (48.4) | 36 (94.7) | 46 ( 100) | 1.471 (0.650,3.327) | 0.35 |
|  | No* | 20 (45.5) | 19 (59.4) | 0 | 1 (20.0) | 34 (23.3) | 32 (51.6) | 2 ( 5.3) | 0 |  |  |
|  | Total | 44 (46.8) | 32 (52.5) | 7 (43.8) | 5 (29.4) | 146 (48.5) | 62 (49.2) | 38 (50.0) | 46 (46.5) |  |  |

NoID: Normal (IQ >70); MID: Mild intellectual disability (IQ 51-70) M-PID: Moderate to profound intellectual disability (IQ 36-< 20)

*Percentages are calculated from the total number of patients with yes and no answers.

#P value calculated from chi-square to test association between categories of intellectual disability (NoID, MID and M-PID) and presence of respective TAND manifestation.
